# University Curricula in Evidence-Informed Decision Making and Knowledge Translation: Integrating Best Practice, Innovation, and Experience for Effective Teaching and Learning

**DOI:** 10.3389/fpubh.2019.00313

**Published:** 2019-12-03

**Authors:** Nasreen S. Jessani, Lynn Hendricks, Liesl Nicol, Taryn Young

**Affiliations:** ^1^Division of Epidemiology and Biostatistics, Department of Global Health, Faculty of Medicine and Health Sciences, Centre for Evidence-Based Health Care, Stellenbosch University, Cape Town, South Africa; ^2^Bloomberg School of Public Health, Johns Hopkins University, Baltimore, MD, United States; ^3^Qualitative Inquiry Group, Faculty of Social Sciences, KU Leuven, Centre for Sociological Research, Leuven, Belgium

**Keywords:** evidence-informed decision-making, knowledge translation (KT), public health, curricula, teaching, adult pedagogy, higher education, facilitation

## Abstract

As attention to Evidence Informed Decision Making (EIDM) and Knowledge Translation (KT) in research, policy and practice grows, so does a need for capacity enhancement in amongst evidence producers and evidence users. Recognizing that most researchers enter the professional sphere with little or no appreciation of the importance and power of EIDM, the Centre for Evidence-based Health Care at Stellenbosch University, South Africa, spearheaded the development and accreditation of a foundational course titled Evidence-Informed Decision making: The Art, Science and Complexity of knowledge translation. The curriculum draws on the principles of adult learning and effective teaching that includes integrating seven key aspects: (1) extraction of intuitive and tacit knowledge (2) autonomous knowledge generation (3) diverse perspectives (4) learning by doing (5) peer-support and critique (6) facilitator coaching and (7) constant reflection. In this paper, we reflect on these techniques in enhancing understanding and utilization of KT in advancing EIDM. The in-person short course has been offered 5 times since its launch in September 2017 with attendance by ~85 senior researchers and government officials—each of whom left the workshop with three completed outputs: a stakeholder matrix, an engagement strategy for their chosen stakeholder and a plan for evaluating the impact of their KT strategy. Interest in the course has grown considerably: (a) Requests from local institutes of research for dedicated training to their staff; (b) Incorporation into international program partner capacity enhancing strategies; (c) Publication of a book chapter designed using course content; (d) Adaptation and utilization of the templates and tools as teaching resources (e) Informing organizational stakeholder engagement strategies (f) Adaptation of the modules for conference capacity building workshops. In summary, designing courses that take into consideration adult principles of learning is not a new concept. However, effective delivery of such courses is still nascent. We found that integrating the seven aspects mentioned above, including researchers together with decision-makers in the workshops, and having an experienced facilitator is critical for effective learning. Enhancing knowledge and skills “just in time” rather than “just in case” has demonstrated increased potential for immediate relevance, uptake and sustainability.

## Introduction

For countries to achieve universal coverage and equitable access to health care for their populations, difficult decisions regarding the most effective use of limited resources, against the backdrop of competing political priorities, need to be made. Given the inherent complexity of such decision-making, health policy and practice decisions can be greatly facilitated if (a) rigorous, robust, relevant, and reliable evidence is available (1–4), (b) decision-makers value and consider evidence in their policy and practice deliberations (5–9) (c) a trusting, respectful and enduring engagement between evidence producers and decision-makers exists (6, 9, 10).

While there are individuals and institutions that are valiantly addressing the first need above, the greater challenge lies in the latter two points giving rise to the field of Knowledge Translation (KT). While the definition of KT has evolved over time, we refer to the prevailing definition that “Knowledge Translation is defined as a dynamic and iterative process that includes synthesis, dissemination, exchange and ethically-sound application of knowledge to improve the health of [the population], provide more effective health services and products and strengthen the health care system.” (11).

KT approaches have been described oftentimes by the actor initiating engagement and the direction of the engagements (12) and comprise 4 models: “producer-push” whereby researchers disseminate results of their studies to perceived and actual users; “user-pull” describes instances when decision-makers actively seek evidence leading to demand-led research; “exchange” efforts recognize collaborative linkages and interactions between producers as well as users; and “integrated” models refer to the added role of a knowledge translation platform or intermediary.

Evidence-informed Decision Making (EIDM) is therefore closely linked to the efforts of KT in that EIDM is “the process of distilling and disseminating the best available evidence from research, practice and experience and using that evidence to inform and improve public health policy and practice” (13).

### The Impetus for a Course in EIDM and KT

As attention to EIDM and KT in research, policy and practice has grown, so has the need for capacity enhancement in relevant knowledge, skills and activities amongst evidence producers (e.g., researchers) and evidence users (policymakers, practitioners, media, non-governmental organizations, Civil society organizations, the public etc.). Various scoping studies have revealed an encouraging response to offering KT specific curricula. For instance, a global scoping study of KT curricula commissioned by International Development Research Centre (IDRC) Canada, in 2008 (14) identified 123 certificate, diploma and post-graduate level KT offerings. However, the differences in the distribution between Low and Middle-Income countries (LMICs) and High-Income Countries (HICs) was marked: only 6 (5%) KT curricula were created by LMICs for LMICs and 11 KT curricula were produced for/conducted in LMICs in partnership with HICs. The rest (86%) were all focused on HICs. An internal review of “courses” identified through internet web searches in 2014 by the Centre for Evidence-based Health Care (CEBHC) at Stellenbosch University in South Africa (15) identified 17 offerings that fit the search criteria of Knowledge Translation courses globally. Fourteen of the 17 courses were offered in HICs: Canada (6), UK (3), Australia (2), US (1), Netherlands (1), and Kosovo (1). EVIPNet Europe initiated a mapping of university-embedded KT curricula in 2015 (personal communication), with a focus on HICs (Europe and the Americas). The results of the mapping study are not yet available, however. While the two scoping studies mentioned above employed different search and identification criteria, courses aimed at researchers often aligned with the objectives of KT in general to:

- Enrich understanding and appreciation of the user context
- Encourage trusting relationships between researchers and research-users
- Enhance capacity of researchers to package, share, and communicate research plans as well as results in a variety of ways relevant to research-users.

While such courses were offered by institutions in a variety of formats, the IDRC study demonstrated that of the 75 offerings by universities at the time—none were African (14). To fill this gap, the IDRC supported the creation of a KT course at the Kenya Methodist University in 2011. The growing interest in KT in LMICs since then has been demonstrated by the plethora of one-off workshops through donor-funded initiatives or conferences in several countries—oftentimes with differing KT foci. Unfortunately, we were unable to find public evaluations of any of the listed courses that might have further informed our planned course in this area. The concerns about sustainability, integration and mainstreaming of KT-relevant skills and capacities amongst the next generation of researchers remain important, topical issues that urgently need to be addressed.

In response to the above, the Centre for Evidence-based Health Care (CEBHC) at Stellenbosch University, South Africa spearheaded the development and accreditation of a foundational course titled *Evidence-Informed Decision making: The Art, Science and Complexity of knowledge translation*, followed by modules designed for engagement with stakeholders.

Given that KT is a large and evolving field, the required skills set is equally large in breadth and depth. The course is, therefore, designed to permit participants to leave the course with not only a better understanding of the theory and science of KT but also practical tools to navigate the complexity of its implementation in different contexts.

## Pedagogical Framework(s), Principles, and Competencies/Standards Underlying the Educational Activity

“Tell me, and I will forget. Show me, and I may remember. Involve me, and I will understand.” *Confucius*

We believe that educators need to be facilitators of learning (16)—“guides on the side”—rather than teachers or “sages on the stage” in the traditional definition. Skills in facilitation of such learning have been shown to be important elements of the learning experience (17, 18). To maximally inspire the learning experience of the course content we employed principles of Piaget's *constructivism*, Papert's *constructionism* (19) and Vygotsky's *socio-constructivism* (20) in the design and delivery of the course. As such the overarching pedagogical principles upon which the design and delivery of the course was based, were that learning is incremental, “developmental, experiential, and interactionist” (21) and that participants should “own their own learning” (22).

Mezirow's (23) perspective of Transformative Learning Theory (TLT) posits that adults learn through “aha moments.” As such, “*Discovery*” sessions or “a-ha moment activators” were applied as initiating activities for each topic in order to facilitate the learning process and to explore participants' tacit knowledge and perspectives of key concepts before formally presenting core course materials in each session. We also included deliberate opportunities for facilitated dialogue with peers in order to foster deeper reflection, critical thinking and reasoning (23, 24) among course participants.

Recognizing that researchers, policy makers, and practitioners are not *tabula rasa*s, or blank slates, we expected each participant to approach the course with their own mental models for KT and their own expectations of the value, content, and strategies for effective outputs and outcomes. This understanding, together with Heifetz et al.'s (25) “balcony and dance floor” analogy for adaptive leadership, inspired a course design that encouraged experiential sharing and discussion, that embedded opportunities for zooming in and out of individual projects.

The CEBHC course was therefore designed in a way to dovetail the science and art of KT by drawing on principles of adult learning and capacity strengthening that integrates 7 important aspects: (1) extraction of intuitive and tacit knowledge (2) autonomous knowledge generation (3) diverse perspectives (4) learning by doing (5) peer-support and critique (6) facilitator coaching and (7) constant reflection.

Hence, the course draws upon tools that encourage participants to “own their own learning” by actively being involved in a process of meaning and knowledge construction as opposed to passively receiving information. In Stenhouse's (22) definition, this would reflect “curriculum as process,” while still encompassing elements of knowledge transmission as well as output generation.

### Aim of the Paper

In this paper we describe our experience with the design and delivery of a short course in KT, aimed at LMIC participants that incorporated foundational techniques of constructivism and adult pedagogy to enhance the understanding and utilization of KT in advancing EIDM. In particular we:

- Describe the approach and content of the course curriculum
- Provide details on how we integrated key pedagogical principles from across history and disciplines as a means to deliver the course material.
- Share our reflections and that of course participants, on the implementation process and the short-term impact of the course.

## Learning Environment (Setting, Students, Faculty); Learning Objectives; Pedagogical Format

### Learning Environment

#### Setting

The course was offered over 2 days using a face-to-face approach. An outline of the course agenda is provided in Annex I. The interactionist principle of learning was engendered by limiting the course to 20 participants and seating participants in groups of 4 to 6 around circular tables. This ensured an intimate and interactive setting.

#### Students

There was no restriction on participants in terms of previous qualifications or seniority. The short course was open to anyone keen to learn more and/or contribute to the learning on this topic and therefore junior researchers, tenured professors, practitioners, decision-makers etc. were all welcome. Each offering of the short course was advertised across the country through social media, emails, networks, and course alumni.

#### Faculty

Faculty members at Stellenbosch University developed the course content, the design as well as the delivery of the course. They also served as facilitators for the various offerings.

### Learning Objectives

The aim of this course was to deliver KT-specific information, tailored to participants from LMICs, using foundational techniques of constructivism and adult pedagogy to enhance the understanding and utilization of the KT information. Specific learning objectives of the course are outlined below, in Box 1.

Box 1 **Specific learning objectives of the course.**

At the end of the foundational course, participants are expected to be able to:

1. Appreciate opportunities, challenges and nuances around the science, art, and context complexity of KT
2. Translate appreciation for the various actors, stakeholders and their roles in the KT continuum into rigorous stakeholder analyses
3. Select and apply various KT strategies and tools appropriately
4. Consider and incorporate time, budget, HR and skills into KT plans
5. Incorporate deliberate measurement and metrics of research uptake and use
6. Design a draft KT and stakeholder engagement strategy

### Pedagogical Format

As mentioned earlier, the overarching pedagogical principle upon which the design and delivery of the course was based, was that learning is incremental, “developmental, experiential and interactionist” (21) and that participants should “own their own learning.”

Each day began with energizing ice-breakers intended to minimize hierarchical beliefs and behaviors (between junior researchers and professors or researchers and clinicians/policy makers) and encourage participation, discussion, networking, and interactionist learning.

The course was divided into five key topics in line with the steps required to design a comprehensive KT and engagement strategy. These include: an overview of key concepts relevant to KT and EIDM, people and power, tools and strategies, resource considerations, and monitoring and evaluation. Each session comprised of three main components: *Discovery* (the “aha” moment), *Concept Introduction* (didatic) and *Concept Application* (practical).

#### Discovery

Interactive polls were used to understand participant values, perspectives, tacit knowledge, and assumptions. For the interactive polls we used Poll Everywhere (26), a web-based program that can be integrated directly into presentation software in order to provide real-time poll results. Poll questions included voting on various EIDM beliefs and myths, interpretation (and consequent reporting of data) etc. This posed some challenges in situations when there was unreliable internet or electricity supply. Flipcharts for the polls and voting were used as optional backups in such cases.

The simulated role-play was designed for the session on stakeholder engagement was a first step in achieving objective 2 (see Box 1). We adapted an existing case study to replicate a community meeting in a hypothetical district suffering from maldistributed health services (27). This exercise encouraged course participants to step out of their comfort zones and embody various personas (for example midwives, the district chief, patients) from the district, each with a different perspective of the health care disparities experienced, who were likely to be impacted by research and/or policy decisions proposed in the case-study. Observers and role-play participants reflected on and critiqued the simulation leading to questions about power, coalitions, roles of researchers, roles of decision-makers, values, modes of engagement, and conflict management. Participants therefore began to demonstrate appreciation of the various actors, stakeholders and their roles in the KT continuum.

During the “engagement proposal,” course participants were presented with a matching game which aimed to elicit participants' understanding of modes and methods of engaging with stakeholders.

Participants were each assigned a specific target audience (i.e., researchers, media stakeholders, policymakers, practitioners, or members of the public). They then matched the most appropriate engagement tools and strategies, from the list of 29 provided to them, with their assigned audience. This was first done independently and then with other participants assigned to the same audience in a think-pair-share exercise. The objective of the former strategy was to permit participants to judge for themselves what made sense; the objective of the latter step was to expose participants to potentially different choices by peers that may have been influenced by personal mental models, assumptions, experiences and contexts. Where there were differences in choices, participants were challenged to convince the other of their chosen tools and strategies. During the post-discovery discussion participants reflected on questions such as “What did I discover?” and “What affected the differences in my opinions with my peers?” This exercise intended to introduce participants to a variety of KT strategies, tools and their appropriate uses as well as enhance appreciation of tailoring strategies to a specific audience. They were key aspects related to objective 3 and 4 (see Box 1).

The “gallery walk” consisted of five poster size scenarios that outlined an engagement strategy; each for a different audience. Participants had 3 min to discuss how they would evaluate the impact of the engagement and knowledge translation strategy outlined in each poster before moving onto the next one in the gallery. This was the beginning of introducing concepts relevant to objective 5 (see Box 1). After a complete rotation, a group representative reported back to all participants on the poster they were currently stationed at, prompting discussion between all participants for all scenarios. Using their tacit knowledge of indicators and their new learnings of KT, participants began to connect the constructs and deliberate appropriate choices for indicators on research uptake and use. Questions such as “which indicators are critical?” and “how do you attribute change to your activities?” were important to deliberate on.

Each discovery session was followed by a reflection activity, during which the course participants explored diverse perspectives on the learnings experienced during the discovery activity. This assisted with engendering the principles of autonomous knowledge generation and experiential learning.

#### Concept Introduction

The discovery sessions led to enhanced interest about the topics that we complemented with formal, structured presentations on the concepts that emerged in the discussions and deliberations prior. We had a total of 5 presentations to introduce the concepts, each focusing on a specific aspect of the course but building upon the previous materials. We interwove didactic approaches throughout the short course to concretize the value, utility, scope, and structure of the concepts across objectives 1 to 6 (see Box 1). This approach was used in parallel with more interactive engagement of participants to enhance a learning-by-doing philosophy and engender more developmental learning. These were, once again, speckled with opportunities for discussion, debate and reflection.

#### Concept Application

The first step in “owning one's own learning” is to incorporate learned concepts into practical activities that are meaningful to each individual. To facilitate this, participants were requested to bring to the course, a specific research result or policy-relevant issue they wished to share with a wider audience. Consequently, each participant designed, planned and constructed three outputs by the end of the course: a stakeholder map for their selected projects or research studies (linked to objective 2, see Box 1), a draft engagement strategy for one particular stakeholder considered a priority (linked to objectives 3, 4, and 6, see Box 1), and a plan for evaluating the impact of their KT strategy including a variety of indicators for each of the activities outlined in the KT strategy (Objective 5).

Using a template created by the lead author, the engagement strategy for each stakeholder followed the steps from the KT engagement framework (28): the purpose for engagement, the message, the medium/forum, the messenger, the timing, the resources required and indicators to measure the effectiveness of the planned KT and engagement strategy. Detailed steps and references for the content of each have been documented elsewhere (29).

By encouraging participants to create products based on pre-identified topics relevant to their work place, we employed the principle of “just in time” learning (JIT). JIT learning creates immediate value to participants in contrast to learning topics “just in case” (JIC) that may (or may not be) relevant or useful in the future. With JIT learning, participants are able to strategically align their new knowledge to their work, implement it immediately, engage with facilitators and peers to reinforce it, and increase their chances of retaining it.

Throughout the course, deliberate opportunities for reflection, feedback and support from facilitators as well as peers were carved into the agenda to engender the importance of learning by interacting (21).

Box 2 shows the link between the various Discovery, Concept Introduction, and Concept Application sessions and the specific learning objectives of the course.

Box 2 **Pedagogical approaches for delivering on the KT course objectives.**

**Table:** 

**Course learning objectives**	**Pedagogical approach**
1. Appreciate opportunities, challenges and nuances around the science, art and context complexity of KT	**Session 1** Discovery: *Poll position*—interactive polls with questions on various EIDM beliefs and myths, interpretation (and consequent reporting of data) etc. Concept introduction: Presentation on *Art, Science and Complexity: Overview and introduction to EIDM and KT*
2. Translate appreciation for the various actors, stakeholders and their roles in the KT continuum into rigorous stakeholder analyses	**Session 2 and 3:** Discovery:*Rock and Role* (role play and simulation) Concept introduction: Presentation on *People and Power* Concept Application: Map the power and interest of stakeholders particular to the research issues (advocates/adversaries)
3. Select and apply various KT strategies and tools appropriately	**Session 4 and 5:** Discovery: The Engagement proposal A matching game to identify which KT tools and approaches are appropriate for engaging different audiences followed by a Think-Pair-Share exercise Concept introduction: Presentation on *Mind the gap* Concept Application: Design a KT engagement strategy for a chosen stakeholder using appropriate tools and approaches
4. Consider and incorporate time, budget, HR and skills into KT plans	**Session 6:** Concept introduction: Presentation on *Strategic Opportunism: Resource considerations for a decision-maker engagement strategy* Concept Application: Map needed resources to the KT engagement strategy designed in previous session
5. Incorporate deliberate measurement and metrics of research uptake and use	**Session 7 and 8:** Discovery: *Match made in heaven* A gallery walk, how would you assess the impact of the presented KT strategy scenarios Concept introduction: Presentation on *What gets measured, gets treasured* Concept Application: Outline a means of evaluating the various tools and approaches embedded in participants' KT engagement strategy
6. Design a draft KT and stakeholder engagement strategy	The concept application across Session 1–8 culminate in a completed draft engagement strategy for one stakeholder that includes approaches, tools, resources and methods for monitoring and evaluating the strategy

### Course Evaluation

The combination of presentations, case examples, expert engagement, peer discussions, and the provision of templates for course deliverables embody the philosophy behind the training with emphasis on completed products for use “just in time” as opposed to “just in case.”

Given that this was a short course offered for professionals outside of any degree requirement, there was no requirement to offer evaluations in the forms of examinations to test knowledge or analyze student learning. However, in the spirit of ensuring the path to mastery we used four methods (outlined below in Box 3) to evaluate the extent to which we achieved the specific learning objectives of the course (see Box 1). Two evaluation methods were embedded within the course with one administered at the end. This is similar to methods used in approaches to Problem-Based Learning (PBL) (30, 31).

Box 3 **Methods for evaluating participant learning and course utility.**

1. Ensuring that the participants actually produced several outputs as a result of the training (stakeholder analyses for objective 2, create a fully resourced stakeholder engagement and KT strategy for Objectives 3 and 4 and 6, and develop indicators to measure the various activities outlined in the KT strategy for Objective 5) throughout the course. Evaluation of the outputs through peer as well as facilitator feedback contributed to enhanced learning and mastery
2. Embedding structured time for reflections and discussions throughout the course on all issues relevant to objective 1–6 so as to provide facilitators with real-time feedback on participant understanding, challenges and perspectives. This allowed for any course correction, immediate clarifications, and peer-peer learning
3. Participant evaluations of the course that asked specific questions with respect to achievement of the specific learning objectives as well as the utilization of the key pedagogical principles that we outline. At the end of each course, participants completed an online, anonymized evaluation form comprised of Likert scale and open-ended questions (see Annex II). The Likert scale questions assessed participants' satisfaction with the content of the presentations, the facilitators and the organization of the training as well as the effectiveness of the practical, peer, and discovery sessions. Feedback from the evaluation forms led to improvements and adaptations to subsequent offerings of the course
4. Informal feedback and reflections over time with previous participants about the value of the course provided anecdotal evidence of course relevance and utility

Facilitators debriefed after each course offering to reflect on new ideas, challenges to previous ideas, innovative examples, and participant feedback to refine and enhance the content as well as delivery of each subsequent version of the course. The course continues to evolve as a result.

## Results to Date

The in-person short course has been offered 5 times since its launch in September 2017. Majority of the participants were academic researchers from universities or research councils (55%) (Table 1). Most participants (*n* = 71) were from South Africa with nine delegates from other countries (Table 2).

**Table 1:** Distribution of participants by organization.

**Organizational affiliation**	**Number (%) of participants**
Academia	44 (55%)
Healthcare/NGO	29 (36%)
Students	4 (5%)
Government	3 (4%)
Total	80 (100%)

**Table 2:** Distribution of participants by Country.

**Country**	**Number (%) of participants**
South Africa	71
Rwanda	2
Ethiopia	2
Malawi	2
Uganda	2
Germany	1

### Course Outputs

Each participant left the course with three completed outputs: a stakeholder matrix, an engagement strategy for their chosen stakeholder and a plan for evaluating the impact of their KT strategy relative to their chosen stakeholders. Specific templates for designing the KT strategy were developed by the principal author (NJ).In this way the specific learning objective 6 and the pedagogical framework around *Concept application* were met. The design of the course provided opportunity for constant reflection through group discussion, facilitator input and peer feedback contributing to iterative refinement of each of these course outputs.

### Reflections on the Learning Style/Pedagogy

Participants were satisfied with the organization, structure, and facilitation method of the courses.

**Figure 1:**
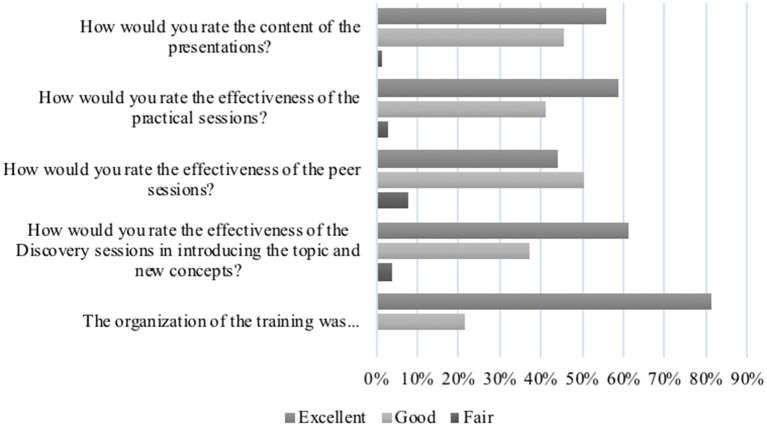
Participant evaluation of the pedagogical approaches to the course (2017–2019).

Figure 1 provides an overview of respondent reflections on the various aspects of the course. None rated any of the aspects as “poor.”

The discovery sessions elicited the most energy and excitement as they sparked the desired “a-ha” moments for participants. They enjoyed the challenge of testing their assumptions and preconceived notions to interrogate a concept before diving into the theory of it. These sessions allowed the facilitators to discern tacit knowledge and assumptions from which to springboard discussions and learning so as to equalize the knowledge base. Participant reflections are noted in the quotes below:

“*[The stakeholder simulation] was a great, creative and exciting session - very different to my usual work.”* (November 2017).

“*[The most enjoyable/useful part of the workshop was] the [gallery walk]discovery session on metrics.”* (March 2018).

The majority of new content was delivered through presentations, complemented by discussions and deliberation as mentioned earlier. Different aspects resonated with participants, depending on their previous knowledge and experience of a topic. However, new ways about thinking of some of the topics was also highlighted:

“*The session on stakeholder mapping was very useful as it provides important frameworks for conducting this highly important step in knowledge translation.”* (September 2017).

Putting learnings into immediate practice allowed participants to reflect on the relevance and realities of some of the theoretical concepts and it also allowed them to leave the sessions with outputs that were immediately useful. Participant evaluations indicate a 97% satisfaction score (Figure 1) for this aspect of the course with reflections on the immediate utility of the products:

“*[The most enjoyable/useful part of the workshop was] the practical session, where you were challenged to apply this to your current work. It was great it was not a theoretical course only.”* (November 2017).

### Reflections on Peer Learning

The power of peer engagement and contributions was evident in the energy that was created when such opportunities arose. In Figure 1, 95% of participants evaluated the peer sessions as either “excellent” or “good.” Participants often got excited about the contributions they were able to make in their peers' outputs and immensely grateful for their peers' contributions to their own work:

*My favorite aspect of the course were the peer sessions. They enabled me to get other people's practical insights on the research project I'd like to undertake. The fact that peers' experiences were so diverse and different from mine created an opportunity to consider other approaches I might not have thought about*. (November 2017).

### Reflections on Facilitation and Content

**Figure 2:**
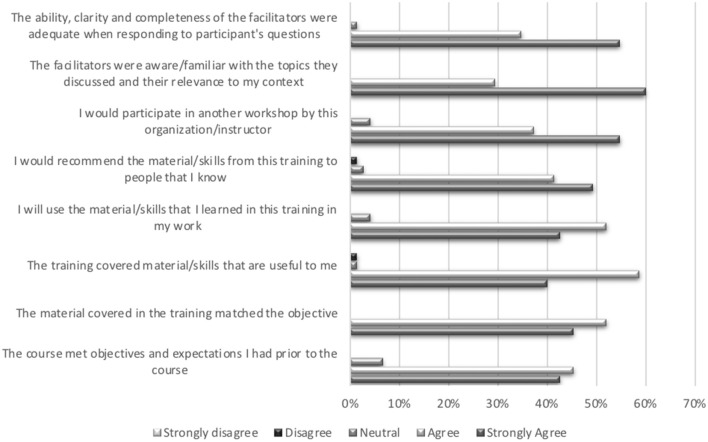
Participant evaluation of the facilitation and content of the course.

Figure 2 indicates participant appraisal of facilitation and content of the course.

Facilitation using the key pedagogical approaches was an important element of the course. We used participant feedback to continue to improve the delivery and content of the course as well as the time allocated to the various sessions. We also ensured that the content of the course was contextually relevant, something that participants contributed to as well as appreciated:

“*I thought the facilitation was grounded and realistic - often at workshops like this, I find people running through their material and formulaic responses without the relevant nuance and flexibility to audience etc. but I felt it was well handled, insightful, and useful.”* (September 2017).

The various forms of our evaluations provided us with positive feedback from participants on the value of incorporating their learnings from the course into their own work environments. Informal conversations with, and impromptu emails from, several participants indicate that they have actively integrated the principles, learnings, and tools into their programs and projects in order to enhance relationships and engagement with their identified stakeholders. The courses have also led to an informal network of colleagues across disciplines sharing their experiences of KT strategy implementation and use.

“*It is almost disturbing to think that this kind of training is not a more standardized tool being used in research settings, because it would save some time in the end. However, I'm glad that I was able to attend and learn skills that would enable me to refocus my workload in a more constructive way.”* (September 2017).

“*This was a great course and needs to be incorporated into academic curricula for all researchers!”* (November 2017).

“*It was interesting to note that the course could be applied to projects at conception stage and completed projects requiring implementation.”* (March 2018).

### Constructive Feedback

The inaugural course was 3 days long. Following constructive feedback from the evaluation forms, to reduce the time dedicated to practical sessions, we scaled the course back initially to 2 and a half days and finally to 2 days. Overall, the value for such an offering was evidenced by interest in the course (or modules within it) growing considerably as shown in Box 4:

Box 4 **Evidence of increased demand for a course on EIDM and KT (2017–2019).**

- Requests from local institutes of research for dedicated training for their staff (South African Medical Research Council, Health Systems Trust)
- Incorporation into international program partner capacity enhancing strategies (Future Health Systems, 2019, Collaboration for Evidence-Based Healthcare in Africa (CEBHA+), 2018, Bill and Melinda Gates Institute for Population and Reproductive Health, 2019)
- Publication of a book chapter formulated using course content (29)
- Adaptation and utilization of the templates and tools as teaching resources (Stellenbosch University Centre for Rehabilitation Studies, Stellenbosch University Centre for Rehabilitation Studies Department of Global Health)
- Informing organizational stakeholder engagement strategies (Cochrane South Africa)
- Adaptation of the modules for conference capacity building workshops (Cochrane South Africa, 2019)

Informal conversations with, and impromptu emails from, several participants indicate that many have actively integrated the principles, learnings and tools into their programs and projects in order to enhance relationships and engagement with their identified stakeholders. The courses have also led to an informal network of colleagues across disciplines sharing their experiences of KT strategy implementation and use.

## Discussion on the Practical Implications, Objectives and Lessons Learned

Designing courses that take into consideration adult principles of learning is not a new concept. However, effective delivery of such courses is still nascent. The strength of this paper is the description of the experience of incorporating key principles of adult pedagogy to design and deliver a short course on Evidence-Informed Decision making and Knowledge Translation, in South Africa, where documented evidence is limited. Results from 5 iterative versions of the course demonstrated that integrating seven key pedagogical strategies (1) extraction of intuitive and tacit knowledge (2) autonomous knowledge generation (3) diverse perspectives (4) learning by doing (5) peer-support and critique (6) facilitator coaching and (7) constant reflection, including researchers as well as decision-makers in the short courses, and having an experienced facilitator are valued elements of effective learning.

Demand for KT knowledge, skills and activities is growing as demonstrated by the requests received as well as by the plethora of KT courses on offer (14, 15, 32). If the next generation of researchers need to be better prepared to engage with a variety of actors and stakeholders (33–35), it is imperative that these skills be embedded into Master and Doctoral training programs which would enhance ownership as well as sustainability of the courses (36). Skills in EIDM KT have been recognized as part of the competencies needed for health professionals and researchers (37–39) but have yet to be designed and integrated into training programs. We therefore promote the enhancement of graduate curricula to match industry needs and equip graduate with the relevant skills and competencies. One may argue that a standalone course such as this one could be one option for embedding such skills. However, students with little understanding of the importance of KT skills may not necessarily opt to take such a course unless required (40). This was clear in our discussions with participants who were seasoned researchers that indicated that they hadn't realized the importance of these skills; suggesting that learning about the utilization aspect of research—and not just the generation of it—should be a fundamental part of research training.

We posit that the skills embodied in this course should be embedded across a variety of graduate programs so that students learn to employ relevant techniques for stakeholder engagement and integrated knowledge translation in whatever research they pursue. We also recommend that the programs should consider employing or adapting the experience of our pedagogical approach in order to deliver knowledge and practical application of these skills. However, this would require architectural accommodations for classes that do not conform to auditorium style lectures (41); it would require all faculty to understand the principles of KT and also embody a “learning paradigm” in contrast to an “instruction paradigm” (42, 43); it would require students to be comfortable with owning their learning (44); and it would require an empathetic atmosphere for students with more introverted dispositions.

While most of the KT courses on offer use a face-to-face approach, there are a few that are delivered online and others that utilize a blended learning approach, incorporating both face-to-face and online learning (14, 15, 32). With constant innovation in teaching and learning, it is possible that courses such as these could evolve into a more blended approach (45) over time or provided entirely online similar to a course on Evidence-Based Health Care (46). However, experience with the intensity of this course as well as the value for the peer interactions and practical aspects, indicate that a blended approach may compromise many of the seven key principles that, together, contributed to the innovation in this approach. While each of the seven principles have already been demonstrated, individually, as effective, we combined them in order to capitalize on their individual effectiveness for enhanced learning. This is the first instance, that we are aware of, where such an innovative approach has been perused and we argue for continued experimentation in this way.

### Limitations and Constraints

The reflections in this paper result from the facilitators' own assessment that was informed by their experiences, their reflections, and feedback from participants. To truly understand the long-term impact of courses such as these, it would be important to design pre-post studies that are able to capture the true learning and retention of skills and knowledge (44). One may argue that other study designs could consider two versions of the course offered simultaneously (47, 48)—one more traditional, the other incorporating these seven key elements—to distinguish empirically whether one is more effective than the other. However, given the fact that the curriculum was designed with evidence of effective strategies, it would seem counter-intuitive and perhaps even wasteful to test whether proven techniques are indeed best-practice. What would be important is to see how best to export these techniques and experiences into other educational arenas. An independent external evaluation of the course over time may be able to determine the combined effectiveness but that was beyond the scope of this paper.

## Conclusion

In conclusion, enhancing knowledge and skills “just in time” rather than “just in case” has demonstrated increased potential for immediate relevance, uptake and sustainability. Therefore, integrating best practice, innovation and experience can greatly enhance effective teaching and learning in the field of KT, and perhaps more broadly.

## Data Availability Statement

The datasets generated for this study are available on request to the corresponding author.

## Author Contributions

NJ and TY conceived the course. NJ and LN designed the curriculum, the tools, and the templates. NJ delivered all the short courses and workshops to date and led the drafting and writing of the manuscript. LH and LN co-facilitated some of the workshops. All authors reviewed and approved the final version.

### Conflict of Interest

The authors declare that the research was conducted in the absence of any commercial or financial relationships that could be construed as a potential conflict of interest.

## Appendix

**Annex I:** Course agenda.

**(Day 1): EIDM/KT, Stakeholder Mapping**	**(Day 2): Decision-maker engagement strategy and research use, M and E**
**Refreshments and Registration**	**Refreshments**
**Session 1**	**Session 5**
**Opening** •Welcome and overview of workshop: •Introduction of participants **Facilitated** •Introduction to the workshop •Discovery 1: **Poll Position** •Discussion: Participant reflections •Presentation 1: **Art, Science and Complexity:** Overview and introduction to EIDM and KT	**Facilitated** •Facilitated: Reflections day 1 **Facilitated** •Round Robin: Participants share their chosen decision-makers and why •Presentation 3: **Mind the gap!** Goals and strategies for engaging with various audiences
**BREAK**	
**Session 2**	**Session 6**
**Facilitated** •Discovery 2: **Rock and Role** •Discussion: Participant reflections	**Facilitated** •Practical: Begin to craft decision-makers engagement strategy •Peer feedback: Shared pairs •Discussion: reflections, common challenges •Practical: revise engagement strategy **Facilitated** •Presentation 4: **Strategic Opportunism:** Resource considerations for a decision-maker engagement strategy
**LUNCH**	
**Session 3**	**Session 7**
**Facilitated** •Presentation 2: **People and Power:** Network/ stakeholder mapping •Practical: Individual network and stakeholder mapping relevant to participant research results •Peer feedback: Constructive reflections on selected stakeholder maps •Discussion: Facilitator observations on stakeholder maps/analyses **Facilitated** •Practical: incorporate facilitator and peer feedback. Finalize maps. Transfer to spreadsheet •Facilitated: Q and A, next steps for finalization of stakeholder analysis	**Facilitated** •Practical: Incorporate resource considerations into engagement strategy •Peer session: Shared-pairs. •Practical: Incorporate peer and facilitator feedback on draft. •Facilitated: Q and A, next steps for finalization of KT Strategy **Facilitated** •Discovery 4: **Match made in heaven**. •Presentation 5: **What gets measured, gets treasured:** Monitoring and Evaluating research uptake and use •**Facilitated** •Discussion: Participant reflections •Facilitated: Q and A, next steps
**BREAK**	
**Session 4**	**Session 8**
**Facilitated** •Discovery 3: **The Engagement Proposal!** •Discussion: Participant reflections	**Facilitated** •Practical: Work on an M and E plan for KT strategy •Peer session: Shared-pairs. Constructive feedback **Closing:** •Facilitated: Wrap up Q and A •Evaluation
**EVENING:** participants incorporate feedback, revise stakeholder maps, choose one decision-making stakeholder to focus on for workshop	**ONGOING:** mentorship from course coordinators and/or peer support TBD

**Annex II:** Participant evaluation form for a course on EIDM and KT (2017–2019).

The organization of the training was…	Excellent	Good	Fair	Poor	
Overall the instructors for the training were…	Excellent	Good	Fair	Poor	
How would you rate the content of the presentations?	Excellent	Good	Fair	Poor	
How would you rate the effectiveness of the Discovery sessions in introducing the topic and new concepts?	Excellent	Good	Fair	Poor	
How would you rate the effectiveness of the practical sessions?	Excellent	Good	Fair	Poor	
How would you rate the effectiveness of the peer sessions?	Excellent	Good	Fair	Poor	
The material covered in the training matched the objective	Strongly agree	Agree	Neutral	Disagree	Strongly disagree
The training covered material/skills that are useful to me	Strongly agree	Agree	Neutral	Disagree	Strongly disagree
I will use the material/skills that I learned in this training in my work	Strongly agree	Agree	Neutral	Disagree	Strongly disagree
I would recommend the material/skills from this training to people that I know	Strongly agree	Agree	Neutral	Disagree	Strongly disagree
I would participate in another workshop by this organization/instructor	Strongly agree	Agree	Neutral	Disagree	Strongly disagree
The course met objectives and expectations I had prior to the course	Strongly agree	Agree	Neutral	Disagree	Strongly disagree
The facilitators were aware/familiar with the topics they discussed and their relevance to my context	Strongly agree	Agree	Neutral	Disagree	Strongly disagree
The ability, clarity, and completeness of the facilitators were adequate when responding to participant's questions	Strongly agree	Agree	Neutral	Disagree	Strongly disagree
What was your favorite or most useful aspect of the course and why?	Open-ended				
What was your least favorite or least useful aspect of the course and why?	Open-ended				
Based on the course what aspects should receive the most attention in subsequent trainings?	Open-ended				
Any other comments…	Open-ended				
